# Supplementary material for: Long-term tracking demonstrates effectiveness of a partnership-led training program to advance the careers of biomedical researchers from underrepresented groups
Source: PLoS One. 2019 Dec 12;14(12):e0225894. doi: 10.1371/journal.pone.0225894 (PMC6907819; doi:10.1371/journal.pone.0225894)
Supplement: S1 File — (PDF) [file pone.0225894.s001.pdf]

2018 Training Program Follow-up Survey: SURP

Introduction

In order to demonstrate the efficacy of the Summer Undergraduate Research Program (SURP), we need to learn about student's academic and career activities. This is a survey about your academic/professional goals and accomplishments. Your willingness to participate in this survey is voluntary. You may choose to answer specific questions or refuse to complete this questionnaire. There is no penalty or loss of benefits to you if you choose to respond only to certain questions or if you refuse to complete the survey. It will take approximately 10 minutes to complete this questionnaire. Thank you for your time and feedback!

2018 Training Program Follow-up Survey: SURP

* 1. Please provide your most current contact information in the spaces provided below.

Full name: [ ]

Preferred name: [ ]

Mailing address (street/city/state/zip): [ ]

Email address 1: [ ]

Email address 2: [ ]

Cell phone: [ ]

Permanent phone: [ ]

2. What is the most recent degree you have earned?

- [ ] BA/BS:
- [ ] MA/MS:
- [ ] MBA:
- [ ] PhD:
- [ ] MD:
- [ ] MD/PhD:
- [ ] Other health degree (DDS, etc):
- [ ] Law degree:
- [ ] Some other degree:

Other (please specify)

[ ]

3. What is the name of the institution where you earned your most recent degree?

[ ]

4. What is your current status?

- ◯ Enrolled in an undergraduate program
- ◯ Participating in a training program or fellowship, e.g. post-baccalaureate program
- ◯ Enrolled in graduate school (e.g., MS, PhD)
- ◯ Enrolled in medical school (e.g., MD, MD/PhD)
- ◯ Enrolled in professional school (e.g., MD, DDS, JD)
- ◯ Employed

5. School/program information:

Institution:

Degree and program:

Expected graduation date:

2018 Training Program Follow-up Survey: SURP

6. Please describe employment (e.g., employer, position, responsibilities):

7. It is important for the program to monitor the progress of students in order to secure future funding; therefore, we would like to know if you applied to graduate, medical, or professional school at any time following your participation in the program?

- ◯ Yes
- ◯ No

8. Have you already reported which schools you applied to in a prior Annual Training Program Follow-up Survey?

- ○ Yes
- ○ No

9. To which graduate, medical, or professional programs did you apply?

Institution:

Degree and program:

Institution:

Degree and program:

Institution:

Degree and program:

Institution:

Degree and program:

Institution:

Degree and program:

Institution:

Degree and program:

10. For which programs did you interview?

11. Which programs offered you admission?

12. Of your offers, which program did you choose?

13. Why did you choose that specific program?

Please only respond to this question if you DID NOT apply to graduate, medical, or professional school(s) following your participation in SURP.

14. What educational or career endeavor(s) did you decide to pursue following your participation in the Summer Undergraduate Research Program?

15. Is there any information or guidance the Summer Undergraduate Research Program could have provided to better help you reach your academic/career goals?

2018 Training Program Follow-up Survey: SURP

16. In the past year, have you received any funding for training in cancer research?

- ◯ Yes
- ◯ No

17. Please choose all that apply:

- [ ] Pre-doctoral Fellowship (R25T, etc.)
- [ ] Post-doctoral Fellowship
- [ ] Diversity Supplement (to RO1, R25, etc.)
- [ ] Other Supplement (F31, F32, etc.)

Other (please specify)

18. Please describe the Fellowship and/or Supplement

Institution:

Type:

Start/End Dates:

19. In the past year, have you received any professional awards or honors? If yes, please list the name(s) of the award(s) and/or honor(s).

20. In the past year, have you given any professional presentations e.g., posters or presentations at scientific meetings? If yes, please provide the name of the event(s), if applicable, and/or the presentation topic(s)/title(s).

21. In the past year, have you been an author or co-author on any peer-reviewed research? If yes, please provide the full citation below.

22. Please describe the ways in which your participation in the Summer Undergraduate Research Program impacted your academic/career goals and interests.

2018 Training Program Follow-up Survey: SURP

Thank you very much for taking the time to complete this survey! Your cooperation is very helpful to the sustained funding of the Summer Undergraduate Research Program hosted by the Fred Hutch. If you wish to remove your name from this email distribution list, please contact Marilyn Drennan at: mdrennan@fredhutch.org
