# Supplementary material for: Long-term tracking demonstrates effectiveness of a partnership-led training program to advance the careers of biomedical researchers from underrepresented groups
Source: PLoS One. 2019 Dec 12;14(12):e0225894. doi: 10.1371/journal.pone.0225894 (PMC6907819; doi:10.1371/journal.pone.0225894)
Supplement: S2 File — (PDF) [file pone.0225894.s002.pdf]

2018 Training Program Follow-up Survey: Graduate Students

In order to demonstrate the efficacy of U54-sponsored graduate training programs and academic courses, we need to learn about student's academic and career activities. This is a survey about your academic/career goals and accomplishments. Your willingness to participate in this survey is voluntary. You may choose to answer specific questions or refuse to complete this questionnaire. There is no penalty or loss of benefits to you if you choose to respond only to certain questions or if you refuse to complete the survey. It will take approximately 10 minutes to complete this questionnaire. Thank you for your time and feedback!

* 1. Please provide your most current contact information in the spaces provided below.

Full name:	
Preferred name:	
Mailing address (street/city/state/zip):	
Email address 1:	
Email address 2:	
Cell phone:	
Permanent phone:	

2. What is the most recent degree you have earned?

- ◯ BA/BS:
- ◯ MA/MS:
- ◯ MBA:
- ◯ PhD:
- ◯ MD:
- ◯ MD-PhD:
- ◯ Other health degree (DDS, etc):
- ◯ Law degree:
- ◯ Some other degree:

Other (please specify)

3. What is the name of the institution where you earned your most recent degree?

4. What is your current status?

- ◯ Enrolled in an undergraduate program
- ◯ Enrolled in graduate school (e.g., MS, PhD)
- ◯ Enrolled in professional school (e.g., MD, DDS, JD)
- ◯ Employed

2018 Training Program Follow-up Survey: Graduate Students

5. School/program information:

Institution:

Degree and program:

Expected graduation date:

6. Please describe employment (e.g., employer, position, responsibilities):

2018 Training Program Follow-up Survey: Graduate Students

7. In the past year, have you received any funding for training in cancer research?

- ◯ Yes
- ◯ No

8. If yes, choose all that apply, listing the institution, type, and start and end dates.

Pre-doctoral fellowship (R25T, etc.):

Post-doctoral fellowship:

Diversity supplement (to TR01, R25, etc.):

Other supplement (F31, F32, etc.):

Other:

9. Which of the following best describes your current professional or research interests? Check all that apply.

- [ ] Health disparities
- [ ] Hispanic health issues
- [ ] Cancer research
- [ ] Border health issues
- [ ] Basic sciences
- [ ] Other

10. In the past year, have you received any professional awards or honors? If yes, please list the name(s) of the award(s) and/or honor(s).

11. In the past year, have you given any professional presentations e.g., posters or presentations at scientific meetings? If yes, please provide the name of the event(s), if applicable, and/or the presentation topic(s)/title(s).

12. In the past year, have you been an author or co-author on any published peer-reviewed research? If yes, please provide the full citation below.

13. Please describe the ways in which your participation on the Fred Hutch/NMSU training program impacted your educational/career interests.

2018 Training Program Follow-up Survey: Graduate Students

Thank you very much for taking the time to complete this survey! We value your input and will use your contributions to continually improve the quality of the training programs hosted by the Fred Hutch/NMSU collaboration.
