# Supplementary material for: Long-term tracking demonstrates effectiveness of a partnership-led training program to advance the careers of biomedical researchers from underrepresented groups
Source: PLoS One. 2019 Dec 12;14(12):e0225894. doi: 10.1371/journal.pone.0225894 (PMC6907819; doi:10.1371/journal.pone.0225894)
Supplement: S3 File — (PDF) [file pone.0225894.s003.pdf]

NMSU U54 Student Survey 2018

Survey

This online follow up is conducted by Dr. Mary O'Connell and funded by the U54 Cancer Grant, a partnership between NMSU and the Fred Hutch. As a participant, we will ask you some questions and ask you to provide information about your educational activities and contributions in the past year. We would appreciate it if you could provide us with the following information, since we need to report our continuing progress to the National Institutes of Health on an annual basis.

Your willingness to participate in this survey is VOLUNTARY. You may choose to answer specific questions or refuse to complete this questionnaire. There is no penalty or loss of benefits to you if you choose to respond only to certain questions or if you refuse to complete the survey. It will take approximately 10 minutes to complete this questionnaire. If you choose not to participate or be contacted in the future please provide us with your name and email address in order to remove them from our mailing list and respond to Question #2.

Thank you for your time and feedback! If you have questions or concerns please call Dr. Mary O'Connell at (575) 646-5172 or email her at moconnel@nmsu.edu.

* 1. Name

First Name

Middle Initial

Last Name

Preferred First Name

2. Current Mailing Address.

Address

Address 2

City/Town

State/Province -- select state --

ZIP/Postal Code

* 3. Email Address. If you only have one email address, please enter it under current email address only.

Current Email Address

Permanent Email Address

4. Cell Phone.

5. Permanent Phone. Leave Blank if this is your Cell Phone.

* 6. Project staff would like to follow-up with you periodically to learn whether you are pursuing graduate studies and/or a science/medical related career. By accepting to proceed you agree to participate in the survey and agree to be re-contacted in future.

- Yes, I do wish to participate in the survey and to be re-contacted in the future.
- No, I do not wish to participate and I do not wish to be contacted in the future.

NMSU U54 Student Survey 2018

Current Status

Please let us know if your currently employed and/or enroll in school.

7. What is the most recent degree you have earned? If you have multiple degrees, please enter them as other.

8. What is your current status?

- [ ] 1. No change, already answered in previous survey
- [ ] 2. Enrolled in an Undergraduate Program
- [ ] 3. Enrolled in Graduate School (e.g. MS, PhD)
- [ ] 4. Enrolled in Professional School (e.g. MD, DDS, JD)
- [ ] 5. Employed
- [ ] Other (please specify)

NMSU U54 Student Survey 2018

Change Education

* 9. Has your education interest changed in the past year? (enrolled in a school or program, changed majors or expected graduation date, etc.)

- ( ) Yes
- ( ) No

NMSU U54 Student Survey 2018

Education Status

Please tell us about your current school or education program. If you don't remember the exact dates, please enter the first day of the month you enrolled or graduated from this program.

* 10. Education Information

Institution Name

Degree

Field of Study

11. When did you enroll into this program?

Start Date

MM/DD/YYYY

* 12. When do you expect to graduate?

Graduation Date

MM/DD/YYYY

13. Which of the following best describes your current research interest? Check all that apply.

- [ ] Health Disparities
- [ ] Hispanic Health Issues
- [ ] Cancer Research
- [ ] Border Health Issues
- [ ] Basic Sciences
- [ ] Other (please specify)

NMSU U54 Student Survey 2018

Changed Employment

* 14. Have you change jobs in the past year?

- ◯ Yes
- ◯ No

NMSU U54 Student Survey 2018

Employment Status

Please tell us about where your currently employment. If you don't remember the exact dates, please enter the first day of the month you started or ended your employment.

* 15. Empolyment Information

Empolyer [ ]

Job Title [ ]

Job Description [ ]

* 16. When did you start work with this employer?

Start Date

MM/DD/YYYY

17. Which of the following best describes your current professional interest? Check all that apply.

- ☐ Health Disparities
- ☐ Hispanic Health Issues
- ☐ Cancer Research
- ☐ Border Health Issues
- ☐ Basic Sciences
- ☐ Other (please specify)

[ ]

NMSU U54 Student Survey 2018

Awards

* 18. Were you presented with an award within the past year?

- ◯ Yes
- ◯ No

NMSU U54 Student Survey 2018

Awards

Please list awards, scholarships, or fellowships received in the past year. For all dates, please enter the first of the month when you were presented the award.

1st Award

19. When were you presented this award?

Date

MM/DD/YYYY

20. Award Infomation

Award

Granting
Institution

Description

2nd Award

21. When were you presented this award?

Date

MM/DD/YYYY

22. Award Infomation

Award

Granting
Institution

Description

3rd Award

23. When were you presented this award?

Date

MM/DD/YYYY

24. Award Infomation

Award

Granting Institution

Description

25. If you have more awards to list, please enter them into the box below.

NMSU U54 Student Survey 2018

Presentations

* 26. Have you presented at a conference within the past year?

- ○ Yes
- ○ No

NMSU U54 Student Survey 2018

Presentations

Please list presentations you authored or co-authored in the past year. If you don't remember the

exact date when you presented or when the conference started, please at least enter the first day of the month you presented.

1st Presentation

27. When did you present or when did the conference for this presentation begin?

Date

MM/DD/YYYY

28. Presentation Infomation

Title

Conference

Location

Co-authors

29. Were you invited to present?

2nd Presentation

30. When did you present or when did the conference for this presentation begin?

Date

MM/DD/YYYY

31. Presentation Infomation

Title

Conference

Location

Co-authors

32. Were you invited to present?

3rd Presentation

33. When did you present or when did the conference for this presentation begin?

Date

MM/DD/YYYY

34. Presentation Infomation

Title

Conference

Location

Co-authors

35. Were you invited to present?

36. If you have more presentations to list, please enter them into the box below.

NMSU U54 Student Survey 2018

Publications

* 37. Have you published, submitted, or are in preparation of a publication within the past year?

- Yes
- No

NMSU U54 Student Survey 2018

Publications

Please list publications you authored or co-authored in the past year. Please add publications you've submitted and are preparing, as well.

1st Publication

38. If Published or Accepted, when will it be release? (Only the first day of the Month is required)

Published Date

MM/DD/YYYY

39. Publication Status

40. Publication Infomation

Title

Full Citation
[APA or MLA
format]

2nd Publication

41. If Published or Accepted, when will it be release? (Only the first day of the Month is required)

Published Date

MM/DD/YYYY

42. Publication Status

43. Publication Infomation

Title

Citation

3rd Publication

44. If Published or Accepted, when will it be release? (Only the first day of the Month is required)

Published Date

MM/DD/YYYY

45. Publication Status

46. Publication Infomation

Title

Citation

47. If you have more publications to list, please enter them into the box below.

NMSU U54 Student Survey 2018

Other Funding Opportunities

Please let us know if you have received funding from other fellowships or scholarships.

48. In the past year, have you received any funding for training in cancer research?

- Yes
- No

49. If yes, choose all that apply listing the institution, type, and start and end dates.

Pre-doctoral fellowship (R25T, etc.)	
Post-doctoral fellowship	
Diversity supplement (to TR01, R25, etc.)	
Other supplement (F31, F32, etc.)	
Other (Travel scholarships, mini grants, other scholarships)	

NMSU U54 Student Survey 2018

Thank you

Thank you very much for taking the time to complete this survey!

We greatly value your input and will use your contributions to continually improve the quality of the programs hosted by the NMSU/Fred Hutch collaboration.
