# Supplementary material for: Long-term tracking demonstrates effectiveness of a partnership-led training program to advance the careers of biomedical researchers from underrepresented groups
Source: PLoS One. 2019 Dec 12;14(12):e0225894. doi: 10.1371/journal.pone.0225894 (PMC6907819; doi:10.1371/journal.pone.0225894)
Supplement: S4 File — (PDF) [file pone.0225894.s004.pdf]

Cancer Knowledge Survey

Preamble Text: This is a survey about your knowledge of cancer, your academic/career goals and your accomplishments. Your willingness to participate in this research study is voluntary. You may choose to answer specific questions or refuse to complete this questionnaire. There is no penalty or loss of benefits to you if you choose to respond only to certain questions or if you refuse to complete the survey. It will take approximately 10 minutes to complete this questionnaire. Thank you for your time and feedback!

Section 1. Cancer Health Disparities

1.1. Over the past two years, my understanding of cancer health disparities has increased.

a. strongly agree; my understanding has improved a great deal
b. agree; my understanding has improved somewhat
c. neutral/no opinion; no change in my understanding
d. disagree; I do not feel any more knowledgeable about cancer health disparities compared to before I started this research program
e. strongly disagree; I am even more confused about this topic compared to when I started this research program
f. not applicable (I have not participated in the NMSU-FHCRC research or training)

1.2. Compared to non-Hispanic white women, how likely are African American women to die of breast cancer?

a. more likely
b. just as likely
c. less likely
d. don’t know

1.3. Compared to non-Hispanic white women, how likely are Hispanic women to be diagnosed with cervical cancer?

a. more likely
b. just as likely
c. less likely
d. don’t know

1.4. Compared to non-Hispanic white men, how likely are African American men to die of prostate cancer?

a. more likely
b. just as likely
c. less likely
d. don’t know

Section 2. Cancer Biology

2.1. Over the past two years of training, my understanding of cancer biology and research has increased.

a. strongly agree: my understanding has improved a great deal
b. agree: my understanding has improved somewhat
c. neutral/no opinion/ no change in my understanding
d. disagree: I do not feel any more knowledgeable about cancer biology compared to before I started this program

e. strongly disagree: I am even more confused about this topic compared to when I started this program
f. not applicable

2.2. Which of the following is the best definition of cancer?
a. uncontrolled division of cells in the body
b. uncontrolled division of cells that can invade other tissues
c. genetic mutations leading to uncontrolled division of cells
d. a mass of abnormal cells, all dividing without normal controls

2.3. How likely is early ovarian cancer to be detected with a screening test relative to the detection of early cervical cancer?
a. more likely
b. just as likely
c. less likely
d. don't know

2.4. How likely is an obese man to be diagnosed with colorectal cancer relative to a non-obese man?
a. more likely
b. just as likely
c. less likely
d. don't know

2.5. Which of the following is the current "gold standard" for evaluating the efficacy of novel cancer treatments?
a. animal studies
b. phase 1 clinical trials
c. phase 2 clinical trials
d. phase 3 clinical trials
e. case-control studies

Section 3. Career Progression

3.1. Please select all of the influences/experiences that apply to you from the list below.
- NMSU/FHCRC summer internship for undergraduate students
- NMSU/FHCRC summer internship for graduate students
- Other summer internship
- NMSU/FHCRC research experience (e.g. research in a NMSU/FHCRC project at NMSU)
- Other academic year research experience (with a specific program e.g. MARC or HHMI, or as independent research)
- Shadowing
- Hospital/clinic work (including e.g. working as a scribe in the ER)
- Academic coursework
- Academic/career advice from mentor/advisor
- FHCRC / University of Washington Cancer Health Disparities course
- NMSU Bio 385: Cancer Biology
- NMSU HLS 461: Health Disparities: determinants and interventions
- NMSU/FHCRC Case Studies Workshop
- NMSU/FHCRC Biostatistics Workshop

- NMSU/FHCRC Cancer Teaching Fellow
- NMSU/FHCRC Grant Writing Workshop

3.2. SurveyMonkey® will then present the participant with the specific experiences that they selected in the prior item, along with this prompt:
Rank the THREE MOST important experiences in terms of shaping your academic and/or professional career so far. Give the most important experience a "1", the 2nd most important experience a "2" and the third most important experience a "3".

3.3. Please list any other important career-shaping or professional development experiences here:

3.4. My NMSU/FHCRC research experience has had a positive influence on my plans for my **continued education**.

a. strongly agree
b. agree
c. neutral (no strong impact)
d. disagree
e. strongly disagree
f. not applicable (no NMSU/FHCRC research experience)

3.5. My NMSU/FHCRC research experience has had a positive influence on my plans for my **future career**.

a. strongly agree
b. agree
c. neutral (no strong impact)
d. disagree
e. strongly disagree
f. not applicable (no NMSU/FHCRC research experience)

Section 4. Demographics

4.1 What is/are your **area(s) of scientific specialization**? (Select **up to two**)

Public Health
Epidemiology
Engineering
Cell Biology
Molecular Biology
Genetics
Biochemistry
Teaching/Education
Clinical Practice
Clinical Research
Other, please describe ________________________________

4.2. What is your gender?

Male Female Do not wish to respond

4.3. What is your race?

White Black Asian Native American more than one do not wish to respond

4.4. What is your ethnicity?

Hispanic non-Hispanic do not wish to respond

4.5. Are you a first generation college student in your immediate family?
Yes No do not wish to reply

4.6. Did you / have you received any Pell Grants while an undergraduate?
Yes No do not wish to respond

4.7. Do you have any disabilities?
Yes No do not wish to respond

4.8. What is your current academic standing? [select one]
___ Undergraduate
___ Working in science having completed a BA/BS degree
___ MS level graduate student
___ Working in science having completed an MS degree
___ PhD level graduate student
___ Post-doctoral Scientist
___ Working outside research/academia, completed PhD degree

___ No longer working in science
___ Do not wish to respond

___ Other, please describe ________________________________
