# Supplementary material for: Long-term tracking demonstrates effectiveness of a partnership-led training program to advance the careers of biomedical researchers from underrepresented groups
Source: PLoS One. 2019 Dec 12;14(12):e0225894. doi: 10.1371/journal.pone.0225894 (PMC6907819; doi:10.1371/journal.pone.0225894)
Supplement: Attachment — Submitted filename: Response_to_reviewers.pdf [file pone.0225894.s006.pdf]

College of Agricultural, Consumer and Environmental Sciences
Department of Plant and Environmental Sciences
MSC 3Q
New Mexico State University
P.O. Box 30003
Las Cruces, NM 88003-8003
575-646-3405, fax: 575-646-6041

Dear Dr. Cesario Bianchi
Academic Editor, PLOS ONE

October 30, 2019

RE: PONE-D-19-21375
"Long-term tracking demonstrates effectiveness of a partnership-led training program to advance the careers of biomedical researchers from underrepresented groups"

Thank you very much for the opportunity to respond to the constructive comments of the reviewers. We attach a table to this letter describing in point by point detail our responses to their concerns and the locations in the manuscript where these changes were made. We were able to respond to all of the concerns, and in the vast majority of cases incorporated the requested change.

The only change we are unable to accommodate is the request for more "evidence" to support our claim that the training programs helped promote UR individuals to complete cancer related doctoral degree programs. We deleted the claim of "evidence" from the discussion and point the reader to the self-reports of past participants and the quantitative results of the tracking surveys.

We have complied with the editorial concerns, our submission is compliant with PLoS One file format names, and styles for headings, and title page format, etc, and we have removed the copyrighted images from our supplemental files.

The results of this study demonstrate that long term tracking ≥10 years at a minimum are necessary in order to fully measure the impact of training programs on the undergraduate and graduate student populations. In particular we document the successes of trainees from underrepresented backgrounds in the technical areas of cancer and health disparities research careers. The results of other training programs are not always reported in the literature, and when published often include only short-term outcomes, admission to graduate programs. Our report covers some individuals for 10 -15 years and can document long-term outcomes of career placement, doctoral research completed, among other features. Overall, our manuscript demonstrates the effectiveness of our program to diversify the biomedical workforce.

This is the first time this manuscript has been submitted for publication anywhere.

Thank you for your assistance.

Sincerely,

Mary O'Connell

Mary O'Connell
Emeritus Regents Professor/Distinguished Achievement Professor

Response to Reviewers Concerns

**Concern**<br>(line # refers to original manuscript)	**Response**<br>(line # refers to tracked changes version)
**Editor**	
ensure ms meets style requirements	We have revised the file names for the supplemental files, followed the stuyle sheet for the title page and corrected styles of headings
Copyrighted images in supplemental files	We have deleted the copyrighted images from the supplemental files.
**Reviewer 1**	
Be consistent throughout the paper with use of the terms "racial and ethnic minority", etc	We now use the term "underrepresented (UR) individuals" and "underrepresented groups."
Lines 57 – 63: Make this 2 sentences; it's a run-on	This has been done (line 69-74).
Lines 71 – 75 How do these 3 major research objectives align with the stated goals listed in the abstract?	The three major research objectives provide outcomes that address the goals of degree completion rates, interest in biomedical research, and increased knowledge about biomedical research among underrepresented students; thus, they align well.
Lines 99 – 101 Were these "social relationships" passive or actively and structurally facilitated? If the latter, please describe and discuss the impact on the student	We have added some sentences (Line 149-153) stating that such social events were both active and structural and had the effect of resulting in friendships and camaraderie.
Lines 116 – 118 hear more about this intensive mentoring and what it consisted of	We added some language about the mentoring activities (line 179-183). All students met with their mentor on a weekly basis and discussed their scientific project(s), their future academic career, scientific papers to be written as a result of their work, and steps to be taken to pursue career goals. In addition, each student met one or two times during the summer with the academic mentor of the entire program and discussed career goals and opportunities as well as any student questions.
Line 136 Use of "We" in first person seems out of place	This has been changed to passive voice (line 203).
Lines 173 – 174 Table 1 Why is mention of "…that have faculty who participate in the Partnership" relevant? This is not clear	This has been clarified (line 256-257): "These are limited to the colleges that have faculty who participate in the Partnership, as the University has some colleges that do not participate in the Partnership."
Table 2<br>List what "UG" means under table	This has been added to Table 2 (line 279).
Discussion<br>In general, the manuscript reports "outcomes" not "evidence"<br><br>no mention of varied degree completion rates	We agree we report the career progression as an outcome. We revised the first sentence of the discussion to reflect this change. (lines 376-377)

among undergraduate and graduate students outlined in Table 3, i.e., potential factors, either self-reported or hypotheses, that contributed to this and how this could be addressed moving forward<br><br>There needs to be more direct evidence provided about how the program actively influenced persistence in biomedical research, specifically in relation to cancer relevant projects	A brief discussion of varied degree completion rates was added (lines 408-414)<br><br>We infer that the training program played a part in that outcome, the only “evidence” is that the participants self-report their perceived value of the training program (line 440-442).
Lines 290 – 292 What does “respectable” mean, and how is “very good” being determined in comparison to what?	We deleted the sentence claiming our results were respectable and noted that graduation rates were not very good, but high. (line 406)
Lines 297 – 298 Further, long-term outcomes take time to mature. For us, it took approximately ten years to assess career progression. This is not an “assessment” as much as a report on outcomes	We removed assess and replaced it with “ascertain the outcome of” as that is what we did. (line 424-425)
Lines 313 – 335 Refer to and leverage information provided here to help support claims made throughout the manuscript related to “evidence”	We agree with the reviewers, this section has been left in the discussion. (line 440-473)
Lines 340 – 350 This information should have been included in the Introduction	We believe it is important to iterate the reasons for including underrepresented individuals in academic fields, especially biomedical fields where participation is lacking. This section summarizes why it is important to conduct this kind of work.
**Reviewer #2**	
General<br>Replace “we” with third person<br><br><br>Replace “its”	 We replaced almost all of the “we” with third person, but kept “we” in one section of the manuscript.<br>We replaced all of the ”its” in the manuscript.
Lines 58-59 needs commas	This sentence is restructured, so commas are no longer needed. (lines 69-70)
Lines 71-75 is confusing, needs rewording for clarity	We have reworded these sentences (line 102-106).
Line 83 needs "primarily" or "predominately" added after "institution"	This change was made (line 113)
Line 86, "forms" to "is".	This change was made (line 117)
Line 94, usually "as follows" would be followed by a colon. May also want to add "First..." for the first described strategy to add clarity to paragraph	We have reworded this section (line 141-142).
Lines 102-103, excessively long. Add "undergraduate and graduate" before "students" on 102 and the clause after colon on 103 is not needed.	This change was made (line 154)

Line 126, "i.e." usually off-set by comma or parentheses, not colon.	This change was made (line 191)
Lines 141-142, too many "then" in process explanation (also appears elsewhere in manuscript. This could be more descriptive and clear.	We have added more description and revised (line 208-213)
Lines 152-156, multiple punctuation styles makes the sentence confusing	Revised this section. (line 229-233)
Line 195, "Individuals" should not be capitalized	This change was made, (line 284)
Line 226-227, table title uses capital letter for each word	We corrected this format (line 318)
Lines 228-230, "who" and "which" should actually be "that" in both cases	We made these changes (line 319-320)
Line 250 "resultant" is awkward in sentence and also the research scientists should be a "resultant" category	We removed the word resultant (line 352).
Line 259 ends with comma	We removed the comma (line 366)
Line 273 "have gone on" to "continued"	This change was made (line 380)
Line 274 needs a comma after "degrees"	This change was made (line 381)
Line 277 should have commas to offset "if not higher"	This change was made (line 384)
Line 294 under line number has weird symbol, maybe a track changes mark	We did not see this symbol in our document
Line 298 "us" to "this project" or other similar term--paper is too familiar	We revised this section to third person (line 424-425)
Lines 313-329 holds good information and examples, but comments would be more appropriate in results than discussion	We agree these comments provide good information. Since thematic analysis was not conducted on them, we consider them appropriately placed in the discussion section.
Line 342 "on" to "of"	This change was made (line 488)
Reference 10, I think "D" is "Disparities" and pages need to be reviewed for proper citation notation	This change was made (line 534-536)
